# Uneventful octreotide LAR therapy throughout three pregnancies, with favorable delivery and anthropometric measures for each newborn: a case report

**DOI:** 10.1186/1752-1947-5-386

**Published:** 2011-08-16

**Authors:** Deeb Daoud Naccache, Adnan Zaina, Zila Shen-Or, Michal Armoni, George Kontogeorgos, Ali Yahia

**Affiliations:** 1 Institute of Endocrinology, Diabetes and Metabolism, Rambam Health Campus and Rappaport Faculty of Medicine, Technion, Haifa, Israel; 2 Department of Pathology, G. Gennimatas Athens General Hospital, Athens, Greece; 3 Department of Medicine, E. Rambam Health Campus and Rappaport Faculty of Medicine, Technion, Haifa, Israel

## Abstract

**Introduction:**

The safety of octreotide use, in its short-acting preparation, in pregnancy is still unclear. This report provides the first documentation of uneventful octreotide LAR use during three pregnancies in a woman with bronchial carcinoid-associated adrenocorticotropic hormone-dependent Cushing's syndrome.

**Case presentation:**

A 25-year-old Arabic woman presented to our emergency department with rapid onset of headache, flaring acne and hirsutism, facial puffiness, weight gain and paroxysmal myopathy, and paranoiac thoughts of rape and sexual intimidation. After undergoing surgical removal of a mass by left lower lung lobectomy, her residual lung disease medical therapy failed. Chronic octreotide LAR injections were initiated as indicated by a positive octreoscan.

Follow-up revealed a long-lasting positive response to octreotide. Avidity of octreotide to somatostatin receptor sub-type 2 was later confirmed by a positive somatostatin receptor sub-type 2 in the resected tumor specimen. Against our instructions, the patient had three spontaneous pregnancies leading to delivery of three full-term healthy children while her octreotide LAR therapy continued.

**Conclusion:**

This case adds more data supporting the potential for the safe use of octreotide and the feasibility of octreotide LAR use during pregnancy, making compliance with the patient's preference not to withdraw octreotide therapy as soon as her pregnancy is confirmed a thoughtful option.

## Introduction

The safety of octreotide use during pregnancy does not lend itself to conducting a controlled prospective study. Hence, such assessment is presently dependent on case reports. Ectopic adrenocorticotropic hormone (ACTH)-dependent Cushing's syndrome associated with bronchial carcinoid is well recognized. Though infrequent, it is the leading etiology (30%) of ectopic, non-pituitary ACTH secretion (EAS) [1]. Currently, the prognosis for patients with bronchial carcinoid EAS is good [1-4], even when it persists or manifests as multiple lesions [5]. This outcome is in contrast to the poor prognosis attributed to this disease in the past [6].

When feasible, surgical removal of the causative tumor is the mainstay of treatment. Medical treatment can bridge the gap until surgery is performed or provide adjunctive long-term therapy to suppress hormonal excess of residual disease.

Medical treatments include blockers of steroid synthesis [7] and somatostatin analogues [8]. In many case reports published during the past decade, somatostatin analogues were routinely discontinued once pregnancy was diagnosed. Of special interest is that these case reports comprised seven pregnant women, five of whom had pituitary acromegaly [9-13], one of whom had nesidioblastosis [14], and one of whom had a thyroid stimulating hormone (TSH)-producing pituitary macroadenoma [15], who had uneventful deliveries concomitant to octreotide therapy throughout all trimesters. Five of these women were treated with the short-acting preparation of octreotide, and in two women octreotide LAR was administered [12,15]. Until recently, only one case report described short-period (one-month) use of a long-acting somatostatin analogue preparation, lanreotide, before it was discontinued at the time of pregnancy confirmation [16]. Herein we present the first case report describing a patient who delivered three healthy babies following three consecutive pregnancies while being treated with octreotide LAR for residual ectopic EAS.

## Case presentation

A 25-year-old Arabic woman presented to the emergency department of our medical facility with rapid onset of headache, flaring acne and hirsutism, facial puffiness, weight gain and paroxysmal myopathy, and paranoiac thoughts of rape and sexual intimidation. Her physical examination revealed pronounced facial acne and hirsutism, oily skin, moon face, buffalo hump, and classical Cushing's syndrome purplish skin striae in the abdominal, axillary, and flank regions. Her blood pressure was 150/90 mmHg.

Table 1 presents the patient's relevant endocrine profile. High-dose (2 mg four times daily) dexamethasone failed to suppress both serum cortisol and urinary free cortisol (UFC) levels. Her serum testosterone, 5-dehydroepiandrosterone sulfate, and 17-OH progesterone levels were within normal limits. Chest computed tomography revealed a 22 mm × 15 mm × 10 mm mass in the upper segment of the left lower pulmonary lobe. No adrenal mass was detected.

**Table 1:** Patient's endocrine-biochemical laboratory tests^**a**^

	Time phase				
Test	Baseline	1 month post-surgery	Recurrence 15 months	Steroid blocker treatment 20 months	Octreotide LAR treatment 102 months
Plasma cortisol, nmol/L*	1700	1193	> 1379	495	219
Urinary free cortisol**	1994	2952	5171	1107	120
Plasma ACTH	21.1†		23.2†	17.3†	10.2††
Urinary 5-HIAA‡	6		8.4		3.7

^a^ACTH, adrenocorticotropic hormone; 5-HIAA, 5-hydroxyindoleacetic acid; *normal range 138 to 690 nmol/24 hours; **normal range 55 to 248 nmol/24 hours; †normal range 4.4 to 17.6 pmol/L; ††normal range 0 to 10 pmol/L; ‡normal range 1 to 84 mg/g creatinine.

She underwent a left lower lung lobectomy. The histopathological examination showed a typical carcinoid tumor without mitotic figures or necrosis and with positive immunohistochemical stains for synaptophysin, neuron-specific enolase, and chromogranin A, as well as strong positive staining for ACTH.

The patient became completely free of symptoms with abnormal, though decreasing, UFC levels. A year and a half after surgery she regained weight. Her physical examination confirmed moon face and re-darkening of previous striae. Her UFC levels were high and remained unsuppressed by either low or high doses of dexamethasone (Table 1).

Computed tomography of the chest and abdomen were normal, as was subsequent pituitary tomography. An indium-111 pentetreotide scan obtained to locate an occult focus of the carcinoid revealed a hot focus in the left lower pulmonary lobe and the upper right mediastinum. Treatment with steroid synthesis blockers was initiated.

Mediastinal and paratracheal histopathology of lymph node material obtained by performing a thoracoscopy showed a metastatic carcinoid. Following treatment with octreotide LAR 30 mg/month, she became symptom-free. Her endocrine laboratory results normalized (Table 1).

Almost three years after surgery, while undergoing octreotide LAR treatment, the patient became pregnant. She refused our recommendation to discontinue octreotide LAR therapy during the first trimester, as is routine [17]. Rather, she insisted on continuing octreotide LAR for the duration of the pregnancy because of its effectiveness in maintaining disease remission. A healthy full-term baby was born (Table 2). Two and three years later, respectively, our patient delivered two more healthy full-term babies (Table 2). All three deliveries were by cesarean section. Octreotide LAR treatment was continued throughout this time period.

**Table 2:** Data regarding patient's three pregnancies

	Mother		Newborn	
**Pregnancy**	**Age at delivery, years**	**Delivery, weeks**	**Weight, g**	**Apgar score**

1	27	41	3010	9/10
2	29	40	3085	9/10
3	31	39	3395	8/9

Recent routine follow-up chest tomography 10 years after the patient's initial presentation revealed normal mediastinal lymph nodes, with permanent post-surgical changes at the basal portion of the left lung. The result of a concomitant test for urine 5*-*hydroxyindoleacetic acid was 6.9 mg/day, which is within normal limits (1 to 7 mg/day).

**Figure 1:**
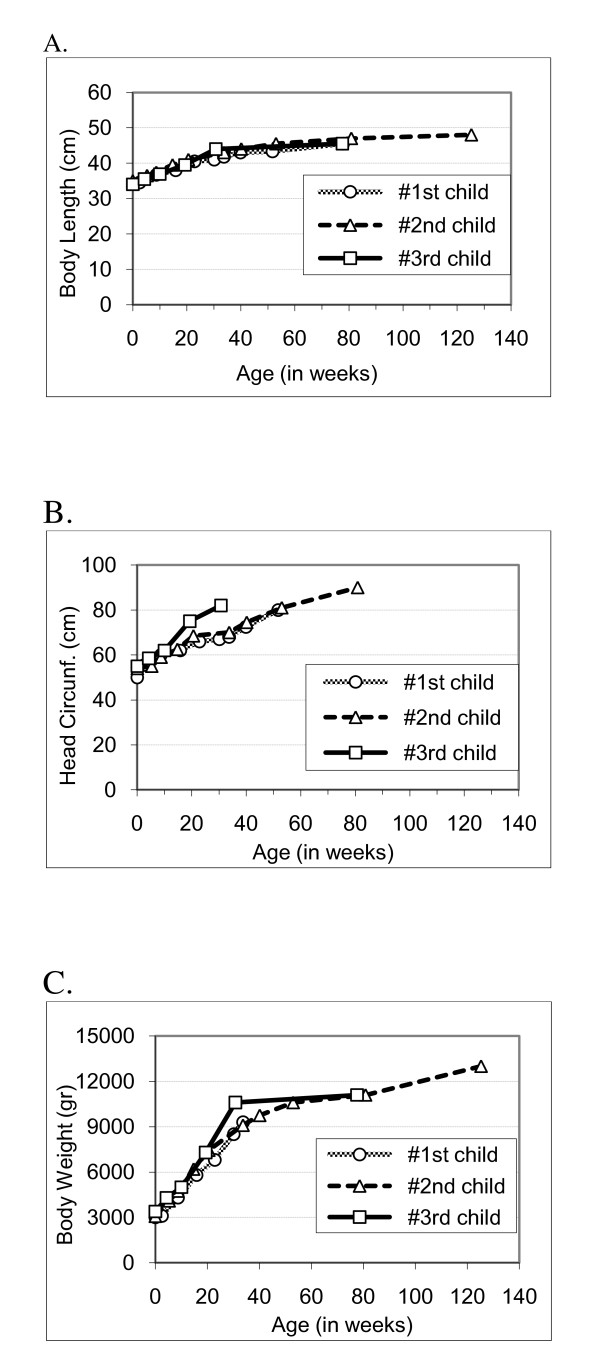
**Anthropometric measures of the patient's three children**. **(A) **Body length. **(B) **Head circumference. **(C) **Body weight.

An immunohistochemistry assay was performed to determine the somatostatin receptor (SSTR) sub-types in the tissue of the original carcinoid in the lung lobe as previously described [18]. The carcinoid tumor tested positive for SSTR types 2A and 2B and negative for SSTR types 1, 3, 4, and 5. The samples taken from the lymph node metastases were inadequate for SSTR immunohistochemistry. Our patient's three babies had normal growth patterns during 128 months of follow-up (Figure 1).

## Discussion

In its short-acting preparation, octreotide has been used safely in humans since 1998. However, its safety during pregnancy is still uncertain. Its administration is usually stopped once pregnancy is confirmed [17]. Information regarding its safety during pregnancy is sparse. We document the safe use of octreotide LAR (its long-acting compound) during one woman's three consecutive full-term pregnancies, all of which were uneventful and yielding healthy babies.

Octreoscan scintigraphy helps select carcinoid patients for somatostatin analogue treatment [19]. A positive octreoscan indicates binding of the analogue for investigation (^111^In-diethylenetriaminepentaacetic acid-D-Phe^1^]octreotide) to SSTR sub-types 2, 3, and 5 [20]. However, 18% of patients with positive octreoscan results do not respond to somatostatin analogues [20]. It is noteworthy that patients who have a good biochemical response or disease stabilization with octreotide treatment stain positive for SSTR2. Those patients who are non-responsive are negative for SSTR2 staining [21]. It seems that a positive response is a result of octreotide binding to SSTR2 [20]. Though not essential for therapeutic decision making, SSTR sub-typing may elucidate our understanding of this rare and heterogeneous disease.

Octreotide crosses the placenta, where it remains stable [12,13,15,22,23]. Previously reported maternal and infant umbilical cord serum octreotide concentrations have been measured, respectively, as 1009 pg/mL vs. 353 pg/mL [12], a range of 4638 pg/mL to 3676 pg/mL vs. 3483 pg/mL [13], 890 pg/mL vs. 251 pg/mL [22], and a range of 2888 pg/mL to 5021 pg/mL vs. 101 pg/mL [15]. Moreover, the half-life elimination time of octreotide approaches 350 minutes in the infant [22] compared to 90 to 110 minutes in adults [24]. Fetal exposure to octreotide due to placental transfer and increased half-life in fetal serum has raised concern about its potential hazard to the fetus [9].

Fetuses seem to be protected from the effects of octreotide. Of primary concern are fetal growth and growth hormone (GH) levels during fetal life. During the third trimester, increasing placental GH production leads to a significant rise in insuling-like growth factor 1 (IGF-1) levels. In this regard, physiological changes in placental GH and IGF-1 were observed during octreotide therapy throughout pregnancy [14]. Similar changes during the last part of pregnancy were reported in a woman with a TSH-producing pituitary adenoma who was undergoing octreotide treatment at the time [15].

Octreotide-driven suppression of GH, however, is tampered because placental SSTRs are mainly of sub-type 4, while SSTR1 remains non-functional as a result of its low affinity for octreotide [25]. In another report, investigators found scanty binding of somatostatin and its analogues to both placental and umbilical cord diverse SSTR1 through SSTR5 [13], which caused the maternal-fetal barrier to sufficiently hamper the functional response of SSTR1 through SSTR5 response to octreotide.

Detection of SSTR2 in the primary tumor of our patient is in accordance with both the effectiveness of octreotide therapy and its lack of detriment to the three fetuses as assessed by their normal post-birth anthropometric measurements.

Seven cases in the literature have reported the safe and effective use of octreotide for the treatment of nesidioblastosis, acromegaly, and TSH-secreting pituitary macroadenoma throughout pregnancy. No deleterious effects on anthropometric measurements during pregnancy [10,14] or breastfeeding under octreotide treatment [9] have previously been observed. Only one case report described low intra-uterine growth (5th to 10th percentile) with no other unusual morphological features [12].

Herein we present the first case report in which octreotide LAR was used to treat carcinoid-associated Cushing's syndrome during pregnancy. Additional case reports are needed to verify the safety of octreotide and octreotide LAR therapy during pregnancy.

## Conclusions

First, our report demonstrates increased evidence for the safety of octreotide treatment throughout pregnancy in addition to that described in the seven previous case reports of safe octreotide therapy, using short- or long-acting preparations, during pregnancy. Second, it supports the effectiveness of octreotide LAR for bronchial carcinoid-associated EAS. Third, it supports the correlation between a good response to somatostatin analogue therapy and the presence of SSTR2 in the diseased target tissue. Fourth, it demonstrates the safe use of octreotide LAR throughout pregnancy and after birth on the basis of the anthropometric data of three babies to the age of two years and older.

## Patient's perspective

"Soon after the disease remission I realized that I resumed my health. I felt powerful enough to challenge the illness and overcome it. Establishing a family was my desire and inspiration. In my opinion having and growing babies, is a clear declaration that I won the combat! I wanted to see them leaving to the kindergarten with bags on their shoulder, exactly the same way other mothers say 'Bye bye' to their children. My father unconditionally supported me; he even stopped smoking for the sake of the first baby's health. The first success with treatment drove me to another two, thank God. All I need is a routine visit to the clinic, and doing some analysis. So what if all I need is a tiny injection every month!?"

## Abbreviations

ACTH: adrenocorticotropic hormone; EAS: ectopic ACTH secretion; SSTR: somatostatin receptor.

## Consent

Written informed consent was obtained from the patient for publication of this case report and any accompanying images. A copy of the written consent is available for review by the Editor-in-Chief of this journal.

## Competing interests

The authors declare that they have no competing interests.

## Authors' contributions

DDN was the attending physician in the out-patient clinic and the attending endocrinologist while the patient was in the hospital. He also drafted and edited the manuscript and obtained the patient's consent and perspective. AZ searched for previous relevant cases in the literature and reviewed and edited the manuscript. ZSO analyzed the laboratory samples obtained throughout the investigation and follow-up period. MA created the figures and reviewed the anthropometric data. GK performed the somatostatin sub-typing in the pathological material. AY was the attending physician while the patient was hospitalized twice during the course of her disease. All authors read and approved the final manuscript.

## References

[B1] Isidori AM Kaltsas GA Pozza C Frajese V Newell-Price J Reznek RH Jenkins PJ Monson JP Grossman AB Besser GM The ectopic adrenocorticotropin syndrome: clinical features, diagnosis, management, and long-term follow-up J Clin Endocrinol Metab 2006 91 371 377 16303835 10.1210/jc.2005-1542

[B2] Aniszewski JP Young WF Thompson GB Grant CS van Heerden JA Cushing syndrome due to ectopic adrenocorticotropic hormone secretion World J Surg 2001 25 934 940 10.1007/s00268-001-0032-5 11572035

[B3] Ilias I Torpy DJ Pacak K Mullen N Wesley RA Nieman LK Cushing's syndrome due to ectopic corticotropin secretion: twenty years' experience at the National Institutes of Health J Clin Endocrinol Metab 2005 90 4955 4962 10.1210/jc.2004-2527 15914534

[B4] Deb SJ Nichols FC Allen MS Deschamps C Cassivi SD Pairolero PC Pulmonary carcinoid tumors with Cushing's syndrome: an aggressive variant or not? Ann Thorac Surg 2005 79 1132 1136 10.1016/j.athoracsur.2004.07.021 15797039

[B5] Aubry MC Thomas CF Jr Jett JR Swensen SJ Myers JL Significance of multiple carcinoid tumors and tumorlets in surgical lung specimens: analysis of 28 patients Chest 2007 131 1635 1643 10.1378/chest.06-2788 17400673

[B6] Shrager JB Wright CD Wain JC Torchiana DF Grillo HC Mathisen DJ Bronchopulmonary carcinoid tumors associated with Cushing's syndrome: a more aggressive variant of typical carcinoid J Thorac Cardiovasc Surg 1997 114 367 375 10.1016/S0022-5223(97)70182-X 9305189

[B7] Farwell AP Devlin JT Stewart JA Total suppression of cortisol excretion by ketoconazole in the therapy of the ectopic adrenocorticotropic hormone syndrome Am J Med 1988 84 1063 1066 10.1016/0002-9343(88)90312-9 3376976

[B8] Hearn PR Reynolds CL Johansen K Woodhouse NJY Lung carcinoid with Cushing's syndrome: control of serum ACTH and cortisol levels using SMS 201-995 (Sandostatin) Clin Endocrinol 1988 28 181 185 10.1111/j.1365-2265.1988.tb03654.x 2844445

[B9] Colao AM Merola Ferone D Lombardi G Acromegaly J Clin Endocrinol Metab 1997 82 2777 2781 10.1210/jc.82.9.2777 9284694

[B10] Mikhail N Octreotide treatment of acromegaly during pregnancy Mayo Clin Proc 2002 77 297 298 11888040 10.4065/77.3.297-a

[B11] Neal JM Successful pregnancy in a woman with acromegaly treated with octreotide Endocr Pract 2000 6 148 150 11421531 10.4158/EP.6.2.148

[B12] Fassnacht M Capeller B Arlt W Steck T Allolio B Octreotide LAR treatment throughout pregnancy in an acromegalic woman Clin Endocrinol (Oxf) 2001 55 411 415 10.1046/j.1365-2265.2001.01304.x 11589686

[B13] Maffei P Tamagno G Battista Nadrdelli G Videau C Menegazzo C Milan G Calcagno A Martini C Vettor R Epelbaum J Sicolo N Effect of octreotide exposure during pregnancy in acromegaly Clin Endocrinol 2010 72 668 677 10.1111/j.1365-2265.2009.03706.x 19769624

[B14] Boulanger C Vezzosi D Bennet A Lorenzini F Fauvel J Caron P Normal pregnancy in a woman with nesidioblastosis treated with somatostatin analog octreotide J Endocrinol Invest 2004 27 465 470 15279081 10.1007/BF03345293

[B15] Blackhurst G Strachan MW Collie D Gregor A Statham PFX Seckl JER The treatment of a thyrotropin-secreting pituitary macroadenoma with octreotide in twin pregnancy Clin Endocrinol 2002 57 401 404 10.1046/j.1365-2265.2002.01549.x 12201834

[B16] de Menis E Billeci D Marton E Uneventful pregnancy in an acromegalic patient treated with slow-release lanreotide: a case report J Clin Endocrinol Metab 1999 84 1489 10.1210/jc.84.4.1489 10199803

[B17] Herman-Bonert V Seliverstov M Melmed S Pregnancy in acromegaly: successful therapeutic outcome J Clin Endocrinol Metab 1998 83 727 731 10.1210/jc.83.3.727 9506716

[B18] Thodou E Kontogeorgos G Theodosiou D Pateraki M Mapping of somatostatin receptor types in GH or/and PRL producing pituitary adenomas J Clin Pathol 2006 59 274 279 10.1136/jcp.2005.026914 16505278 PMC1860351

[B19] Janson ET Westlin JE Eriksson B Ahlström H Nilsson S Öberg K [^111^In-DTPA-D-Phe^1^]Octreotide scintigraphy in patients with carcinoid tumors: the predictive value for somatostatin analogue treatment Eur J Endocrinol 1994 131 577 581 10.1530/eje.0.1310577 7804439

[B20] Janson ET Gobi A Kälkner KM Öberg K A comparison between the efficacy of somatostatin receptor scintigraphy and that of in situ hybridization for somatostatin receptor subtype 2 messenger RNA to predict therapeutic outcome in carcinoid patients Cancer Res 1996 56 2561 2565 8653698

[B21] Janson ET Stridsberg M Gobl A Westlin JE Oberg K Determination of somatostatin receptor subtype 2 in carcinoid tumors by immunohistochemical investigation with somatostatin receptor subtype 2 antibodies Cancer Res 1998 58 2375 2378 9622077

[B22] Caron P Gerbeau C Pradayrol L Maternal-fetal transfer of octreotide N Engl J Med 1995 333 601 602 10.1056/NEJM199508313330918 7623921

[B23] Caron P Gerbeau C Pradayrol L Simonetta C Bayard F Successful pregnancy in an infertile woman with a thyrotropin-secreting macroadenoma treated with somatostatin analog octreotide J Clin Endocrinol Metab 1996 81 1164 1168 10.1210/jc.81.3.1164 8772594

[B24] Chanson P Timsit J Harris AG Clinical pharmacokinetics of octreotide: therapeutic applications in patients with pituitary tumours Clin Pharmacokinet 1993 25 375 391 10.2165/00003088-199325050-00004 8287633

[B25] Caron P Buscail L Beckers A Estève JP Igout A Hennen G Susini C Expression of somatostatin receptor SST4 in human placenta and absence of octreotide effect on human placental growth hormone concentration during pregnancy J Clin Endocrinol Metab 1997 82 3771 3776 10.1210/jc.82.11.3771 9360539

